# Comparative analysis of developmental outcomes in very preterm infants: BSID-II versus Bayley-III German norms

**DOI:** 10.1371/journal.pone.0318263

**Published:** 2025-01-27

**Authors:** Marlene Hammerl, Martina Zimmermann, Anna Posod, Ulrike Pupp Peglow, Michaela Höck, Elke Griesmaier, Ursula Kiechl-Kohlendorfer, Vera Neubauer

**Affiliations:** Department of Pediatrics II (Neonatology), Medical University of Innsbruck, Innsbruck, Austria; University of California San Diego, UNITED STATES OF AMERICA

## Abstract

**Introduction:**

After the release of the Bayley Scales of Infant and Toddler Development, third edition (Bayley-III), US norms, an overestimation of outcome was observed. But, the conformity between the Bayley Scales of Infant Development, second edition (BSID-II), and the Bayley-III German norms is unknown. This retrospective analysis aimed to compare outcomes of very preterm infants tested with BSID-II and Bayley-III German norms.

**Methods:**

Infants born from November 2007 to July 2018 were included. Exclusion criteria were death or missing outcome. Infants underwent testing with either BSID-II until December 2013 or Bayley-III from January 2014 onward, at 12 and/or 24 months. BSID-II Mental Developmental Index (MDI) was compared to Bayley-III cognitive score and a combined Bayley-Score (CB-III) consisting of the cognitive and language composite score. BSID-II Psychomotor Developmental Index (PDI) was compared to Bayley-III motor composite score. Abnormal outcomes were defined as scores <85 (delay) or <70 (impairment).

**Results:**

649 infants were included. At 12 months, the Bayley-III cohort achieved higher scores in all domains compared to the BSID-II cohort (all p<0.05), with lower rates of motor delay in the Bayley-III cohort (p<0.001). At 24 months, only Bayley-III motor composite scores were higher than the BSID-II PDI (p<0.001). Rates of cognitive impairment were higher in the Bayley-III cohort (p = 0.013).

**Interpretation:**

Our findings indicate that the Bayley-III German norms effectively identify children needing interventions, particularly at 24 months corrected age. This supports both clinical application and scientific comparability with the BSID-II.

## Introduction

Preterm birth is associated with an increased risk of poor neurological outcomes compared to term birth [1]. A reliable and valid tool to test cognitive, language and motor skills is essential to ensure optimal support for children with developmental deficits. Further, standardised assessments are important for scientific issues, in particular regarding the comparability of cohorts. However, these tests show an increase in IQ scores over time, attributed to the so-called Flynn effect, which reflects societal and environmental changes rather than actual improvements in intelligence [2]. Consequently, children in need of support may be overlooked if the same tests are used over extended periods. Therefore, it is necessary to regularly revise and update these assessments.

The Bayley Scales of Infant Development (BSID) is a standard test for assessing cognitive, language, and motor development in early childhood. With the second edition of the Bayley Scales (BSID-II) a mental developmental index (MDI), which measures cognitive and language development, and a psychomotor developmental index (PDI) were obtained [3]. In 2006, the third edition (Bayley Scales of Infant and Toddler Development, Bayley-III) was introduced, consisting of a cognitive score, a language composite score (expressive and receptive language), and a motor composite score (gross and fine motor function) [4]. A major advantage of the Bayley-III is the separation of the cognitive and language domain. This improves the classification of developmental weaknesses, allowing better tailoring of therapies [5, 6]. However, contrary to the expected drop in scores and simultaneously higher rates of abnormal test results attributed to the Flynn effect, studies have reported higher scores using the Bayley-III compared to the BSID-II. This led to the assumption that the Bayley-III may overestimate the outcome of infants and therefore under-represents the proportion of developmentally delayed or impaired infants [5–8]. This has been attributed to differences in the sampling of the standardisation groups. For the BSID-II, the standardisation sample excluded children with known developmental deficits, ensuring that the norms primarily reflected children without significant developmental impairments. In contrast, the Bayley-III standardisation sample in the United States (US) included a broader group, with 10% of the children having known developmental problems [3, 4]. This inclusion of children with developmental concerns in the Bayley-III sample may result in higher developmental scores for the general population, as the presence of children with developmental delays in the reference group could potentially elevate the average score. In 2014 German norms of the Bayley-III were published. For these norms the sampling approach was based on the methodology of the BSID-II, to ensure the comparability between the two sets of norms [9, 10]. Despite the publication of the fourth version of the Bayley Scales, there remains a notable absence of studies exploring the comparability between the BSID-II and the Bayley-III German norms. As German norms for the Bayley-IV are not yet available, the Bayley-III continues to be used in clinical practice in German-speaking countries. Additionally, given the anticipated utilization of multiple BSID versions in longitudinal studies, it becomes imperative to address this gap in research.

Hence, the aims of the current study were (1) to compare the scores of very preterm infants born < 32 completed weeks of gestational age tested with BSID-II to those tested with Bayley-III using the German norms, and (2) to analyse if there is a difference in the rates of abnormal neurodevelopmental outcomes between these two groups.

## Methods

### Study participants

This study was a retrospective analysis of prospectively collected data conducted in Tyrol, Austria, with approximately 740,000 inhabitants and 7,500 live births per year, including about 70 very preterm infants (< 32 completed weeks of gestational age). All very preterm infants treated at the Innsbruck Medical University Hospital between November 2007 and July 2018 were eligible for the study. Exclusion criteria were death or lack of outcome data at both timepoints (12 ± 3 and 24 ± 3 months corrected age). Children with outcome data available at least at one of the timepoints were included in the study. Data were last accessed for this study on January 22, 2024. During the data collection and analysis phases, the authors had access to information that could identify individual participants. To protect patient confidentiality, the data were pseudo-anonymized.

### Patient characteristics

Maternal and neonatal data were collected during hospital stay and follow-up visits as described before, and defined in S1 File [11].

### Neurodevelopmental follow-up

All very preterm infants were invited for follow-up visits at corrected ages of 12 and 24 months. In the event of non-attendance, a second invitation was sent.

To assess neurodevelopmental outcomes, we used the BSID-II for children born until December 2013 [3]. Despite the availability of the US norms for the Bayley-III at that time, the Austrian Working Group Neonatology and Paediatric Intensive Care Medicine recommended continuing with the BSID-II using US norms and advised that the transition to Bayley-III should occur once the German norms were published. Therefore, from January 2014 onwards, infants were assessed using the Bayley-III German norms [4, 9].

**Fig 1:**
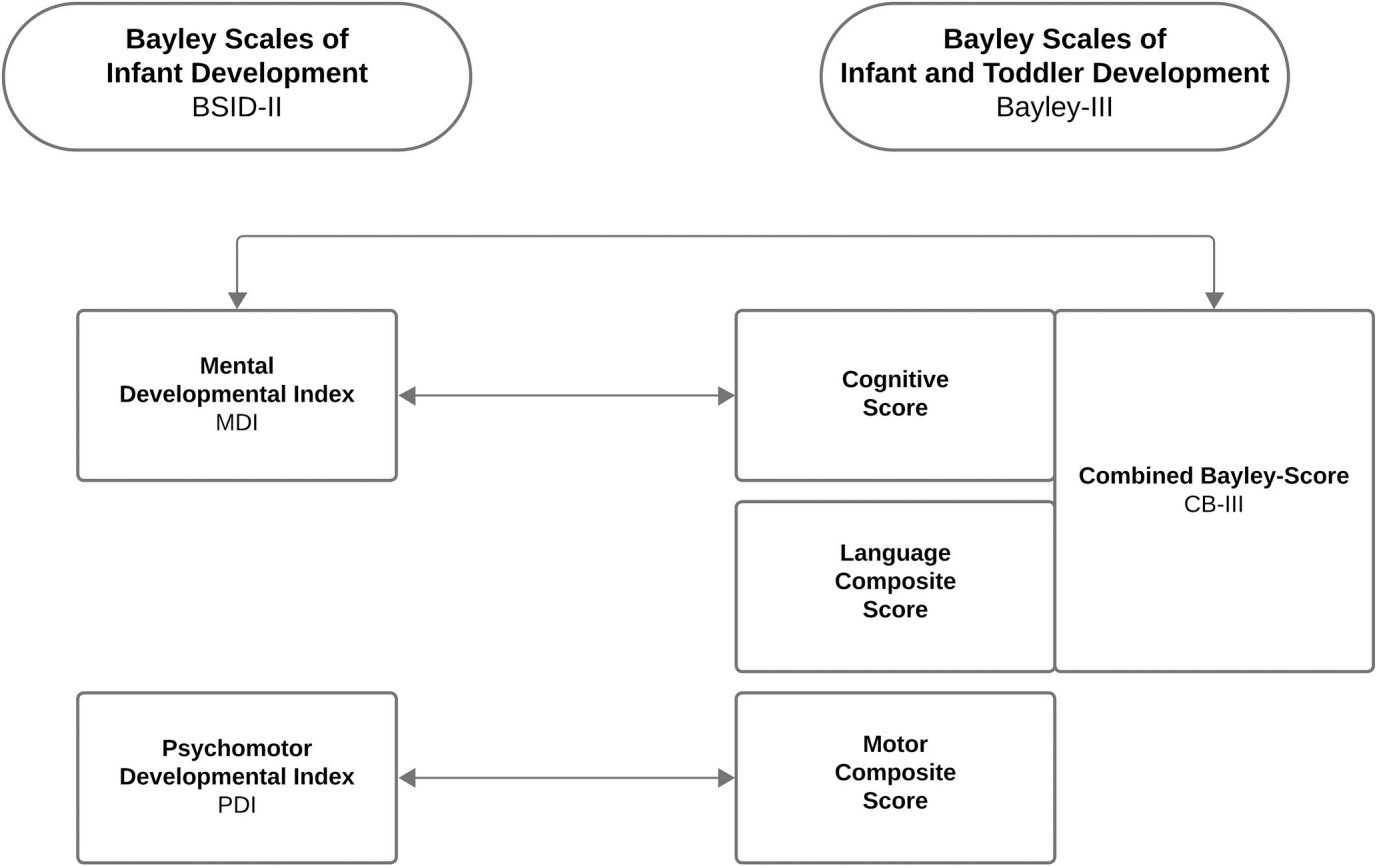
Composite scores of the second and third Bayley Scales editions. Arrows indicate compared scores.

As presented in Fig 1, the BSID-II MDI was compared with the Bayley-III cognitive score and a combined Bayley-Score (CB-III), calculated as the mean between the Bayley-III cognitive score and the Bayley-III language composite score. The BSID-II PDI was compared to the Bayley-III motor composite score. These comparisons align with methodologies used in prior studies investigating outcomes in preterm populations [6, 7, 12]. Despite differences in item composition between the two scales, the mean scores, standard deviation, and cut-offs for identifying developmental delays are defined similarly in both the BSID-II and Bayley-III German norms. This ensures that both assessments aim to identify children with comparable levels of developmental needs, irrespective of the inclusion of new or modified items in the Bayley-III. Abnormal neurodevelopmental outcomes were defined using standard cut-offs: a neurodevelopmental delay was defined as any scores < 85 (> 1 standard deviation [SD] below the mean), and neurodevelopmental impairment as scores < 70 (> 2 SD below the mean) [3, 4]. All tests were performed by experienced psychologists.

### Statistical analysis

Data analysis was performed using R statistics, version 4.2.1. Data distribution was assessed by Shapiro-Wilk-test. For comparison of two independent groups a Mann-Whitney U test was conducted. Comparison of independent categorical data was obtained by using the Fisher’s exact test. To account for differences in neonatal characteristics between children tested with BSID-II and those tested with Bayley-III (5-minute Apgar score, catecholamine use during the neonatal period, presence of patent ductus arteriosus, retinopathy of prematurity grade 3 or 4, duration of CPAP therapy, see Table 1), we adjusted the results for these potential confounders using multiple linear regression for continuous outcomes and multiple binary logistic regression for categorical outcomes. The results of the regression models are presented as B coefficients and Odds Ratios (ORs) with 95% confidence intervals, as appropriate for the models. Data are presented as numbers with percentages and medians with quartiles. Significance threshold was set at 0.05.

**Table 1:** Maternal and neonatal characteristics of the study participants (n = 649).

	all included infants (n = 649)	BSID-II (n = 342)	Bayley-III (n = 307)	p-value
Gestational age (weeks)	30.0 (28.1;31.1)	30.0 (28.1;31.1)	29.9 (28.1;31.1)	0.835
Birth weight (grams)	1260 (980;1550)	1240 (955;1540)	1264 (1000;1554)	0.396
Small for gestational age	58 (9.0)	37 (10.9)	21 (6.9)	0.098
Male	359 (55.3)	184 (53.8)	175 (57.0)	0.430
Multiples	255 (39.3)	134 (39.2)	121 (39.4)	>0.999
Mother achieved university entrance qualification	229 (38.9)	114 (36.9)	115 (41.2)	0.310
Smoking in pregnancy	64 (10.0)	33 (9.8)	31 (10.2)	0.896
Antenatal steroids	564 (91.1)	282 (90.4)	282 (91.9)	0.573
Prelabour rupture of membranes >24 hours	127 (20.6)	58 (18.0)	69 (23.4)	0.111
Vaginal delivery	41 (6.3)	27 (7.1)	17 (5.5)	0.519
Apgar 5’	8.0 (7.0; 9.0)	8.0 (7.0; 9.0)	8.0 (8.0; 9.0)	**0.036**
Surfactant administration	476 (73.6)	243 (71.5)	233 (75.9)	0.212
Catecholamines at birth resuscitation	7 (1.1)	2 (0.6)	5 (1.6)	0.270
Catecholamines during neonatal period	55 (8.6)	40 (11.9)	15 (4.9)	**0.002**
Early onset sepsis	47 (7.4)	25 (7.6)	22 (7.2)	0.880
Late onset sepsis	84 (13.2)	46 (13.9)	38 (12.4)	0.640
Bronchopulmonary dysplasia	158 (24.9)	88 (26.6)	70 (23.0)	0.313
Steroids for bronchopulmonary dysplasia	95 (14.7)	54 (15.9)	41 (13.4)	0.436
Necrotizing enterocolitis	23 (3.6)	11 (3.2)	12 (4.0)	0.673
Patent ductus arteriosus	205 (31.6)	130 (38.0)	75 (24.4)	**<0.001**
High grad retinopathy of prematurity	29 (4.5)	21 (6.2)	8 (2.6)	**0.036**
Duration of ventilation (hours)	2.0 (0.0;12.0)	2.3 (0.0;12.0)	2.0 (0.0;10.0)	0.500
Duration of CPAP therapy (days)	6.0 (3.0; 20.0)	6.0 (2.0; 18.0)	7.0 (3.0; 21.0)	**0.044**
Intraventricular haemorrhage	102 (15.7)	58 (17.0)	44 (14.3)	0.388
Intraventricular haemorrhage grade III-IV	21 (3.2)	13 (3.8)	8 (2.6)	0.506
Periventricular leukomalacia	14 (2.2)	6 (1.8)	8 (2.6)	0.591

Values are numbers (%) or median (25th centile;75th centile). Information on maternal university entrance qualification was available in 588 subjects (90.6%), information on duration of ventilation was available in 599 subjects (92.3%), information on duration of CPAP therapy was available in 604 subjects (93.1%). In all other variables the proportion of missing data was <5%.

### Statement of ethics

The study was approved by the ethics committee of the Medical University of Innsbruck (study No. AN2013-0086 333/4.2). Written informed consent from participants was not required for this retrospective study in accordance with local/national guidelines.

## Results

Of the 763 infants born during the study period, 738 (96.7%) were eligible for the study. A total of 649 infants was included, of whom 342 (52.7%) were tested with the BSID-II and 307 (47.3%) with the Bayley-III German norms.

**Fig 2:**
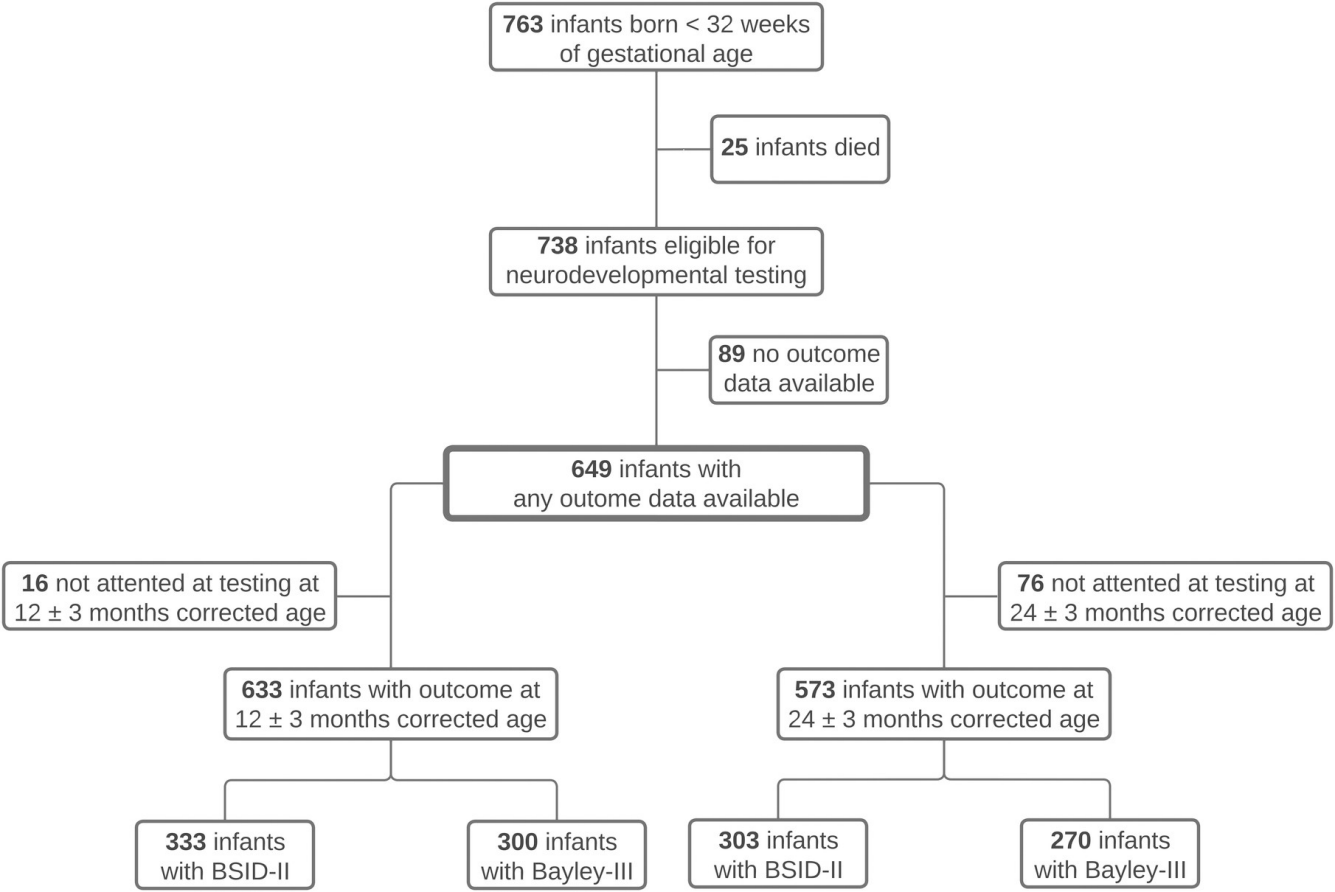
Flowchart of the in- and exclusion procedure of all study participants. Each infant was assessed with either BSID-II or Bayley-III, depending on the birth period.

At 12 months corrected age, neurodevelopment was assessed in 633 (85.8%) of all infants. At this time-point, 333 (52.6%) children were tested with the BSID-II (corrected age: 12 [11;12, range: 9–15] months) and 300 (47.4%) with the Bayley-III (corrected age: 12 [12;12, range: 9–15] months), respectively. At 24 months corrected age, 573 (77.6%) children were assessed. Among these, 303 (52.9%) had scores obtained with the BSID-II (corrected age: 24 [23;24, range: 22–27] months) and 270 (47.1%) with the Bayley-III (corrected age: 24 [24;24, range: 21–27] months). Detailed information on the in- and exclusion procedure is provided in Fig 2.

The included very preterm infants had a median gestational age of 30.0 (28.1;31.1) weeks and a median birth weight of 1260 (980;1550) grams. All patient characteristics are displayed in Table 1.

At 12 months corrected age, univariate analysis revealed significantly higher cognitive scores in the Bayley-III cohort compared to the BSID-II cohort (cognitive score vs. MDI: p <0.001; CB-III vs. MDI: p = 0.003). This finding persisted after adjustment for neonatal characteristics (cognitive score vs. MDI: adjusted p = 0.001; CB-III vs. MDI: adjusted p = 0.011). At 24 months corrected age, cognitive scores showed no significant differences between cohorts in the univariate analysis (cognitive score vs. MDI: p = 0.215; CB-III vs. MDI: p = 0.625). Bayley-III motor composite scores were higher compared to the BSID-II PDI at 12 months (p <0.001; adjusted p <0.001) and at 24 months corrected age (p <0.001; adjusted p <0.001). For detailed information see Table 2.

**Table 2:** Neurodevelopmental outcome at 12 and 24 months corrected age. P-values display significance comparing BSID-II versus Bayley-III German norms.

	BSID-II	Bayley-III German norms	B (95 % CI)	p-value
12 months corrected age				
MDI/cognitive score	101 (90;107), 50–128	105 (90;115), 55–140	0.135 (0.053–0.216)	**0.001** *
MDI/CB-III score	101 (90;107), 50–128	103.5 (94.5;108), 50–133	0.105 (0.024–0.186)	**0.011** *
PDI/motor composite score	89 (81;97), 50–124	103 (92;109), 45–134	0.348 (0.274–0.422)	**<0.001** *
24 months corrected age				
MDI/cognitive score	104 (88;112), 50–134	100 (90;110), 55–145	-	0.215
MDI/CB-III score	104 (88;112), 50–134	101.5 (88.5;113.5), 50–145.5	-	0.652
PDI/motor composite score	100 (91;107), 50–128	106 (96;116), 45–140	0.192 (0.107–0.276)	**<0.001** *

Median (25th centile; 75th centile), range. Comparisons are made between the BSID-II MDI and the Bayley-III cognitive score, the Bayley-II MDI and the Bayley-III CB-III, and the BSID-II PDI and the Bayley-III motor composite score.

*P-values corrected for differences in neonatal characteristics using multiple linear regression.

Correspondingly, rates of cognitive delay were lower in the Bayley-III cohort at 12 months when using the Bayley-III cognitive score (cognitive score vs. MDI: p = 0.019; CB-III vs. MDI: p = 0.084), though this difference was not statistically significant after adjustment for differences in neonatal characteristics (cognitive score vs. MDI: adjusted p = 0.071). The Bayley-III cohort exhibited a higher proportion of children with cognitive or language impairments compared to the BSID-II cohort (CB-III vs. MDI: p = 0.007; adjusted p = 0.013, OR = 2.568). Rates of motor delay were lower in the Bayley-III cohort at 12 months (p <0.001; adjusted p <0.001, OR = 0.301). Complete information can be found in Table 3.

**Table 3:** Rates of abnormal outcome at 12 and 24 months corrected age. P-values display significance comparing BSID-II versus Bayley-III German norms.

	BSID-II	Bayley-III German norms	OR (95 % CI)	p-value
12 months corrected age				
MDI/cognitive score				
<85	40 (12.1)	19 (6.4)	0.542 (0.272–1.039)	0.071*
<70	14 (4.2)	10 (3.4)	-	0.678
MDI/CB-III score				
<85	40 (12.1)	23 (7.8)	-	0.084
<70	14 (4.2)	6 (2.0)	-	0.171
PDI/motor composite score				
<85	116 (34.9)	47 (15.7)	0.301 (0.192–0.461)	**<0.001** *
<70	36 (10.8)	21 (7.0)	-	0.098
24 months corrected age				
MDI/cognitive score				
<85	51 (17.1)	34 (13.1)	-	0.196
<70	15 (5.0)	14 (5.4)	-	0.851
MDI/CB-III score				
<85	51 (17.1)	57 (23.2)	-	0.085
<70	15 (5.0)	28 (11.4)	2.568 (1.241–5.599)	**0.013** *
PDI/motor composite score				
<85	46 (15.3)	39 (14.8)	-	0.906
<70	18 (6.0)	10 (3.8)	-	0.249

Numbers (%). Comparisons are made between the BSID-II MDI and the Bayley-III cognitive score, the Bayley-II MDI and the Bayley-III CB-III, and the BSID-II PDI and the Bayley-III motor composite score.

*P-values corrected for differences in neonatal characteristics using multiple binary logistic regression.

## Discussion

The Bayley Scales of Infant Development are the gold standard test for the assessment of infant development. But, following the introduction of the third edition, concerns arose regarding its sensitivity, as the expected drop in scores typically seen with test adaptations remained absent. An overestimation of outcome by the Bayley-III was soon discussed [2, 5–8, 13, 14]. This is the first study to compare outcomes between the BSID-II and the Bayley-III German norms. While higher scores were indeed observed with the Bayley-III German norms at 12 months corrected age, these differences were clinically insignificant. Importantly, rates of abnormal neurodevelopmental outcomes remained comparable between the two versions, particularly at 24 months.

Social, economic, and demographic characteristics can significantly influence scores [15]. Specifically, the US norms may not accurately reflect the developmental trajectories of children in other regions, such as German-speaking countries, emphasizing the need for population-specific norms to account for these differences [14, 16]. Further, when the Bayley-III was standardised to the US population, 10% of the reference population had known developmental problems [4]. This proportion is higher than expected in a normative sample, resulting in a lower threshold for scoring higher on the assessment [8]. In contrast, both the BSID-II and the Bayley-III German norms excluded children with obvious developmental deficits from their reference populations [3, 9].

The correlation between the US norms and the German norms of the Bayley-III has been documented in previous research, showing that the German norms yielded higher scores in early infancy, but this difference narrowed over time, with only gross motor function remaining higher in the German norms by 24 months compared to the US norms [10]. Our results align with these findings, as infants tested with the Bayley-III German norms achieved higher scores in all domains than children tested with the BSID-II at 12 months corrected age. However, by 24 months corrected age, only the motor composite scores remained higher compared to the previous PDI. Neither the Bayley-III cognitive score nor the CB-III were higher than the BSID-II MDI at 24 months corrected age. While Fuiko et al. found a similar trend of narrowing differences between Bayley-III US norms and German norms with age, our study focuses on a comparison between BSID-II and Bayley-III German norms, providing further context to the observed trends.

In terms of mental development, at 12 months corrected age, the Bayley-III cognitive scores of the German norms were significantly higher than the MDI. This difference may be partly explained by the Bayley-III’s scoring system, which may tend to yield higher cognitive scores for younger infants, as suggested by Fuiko et al. [10]. Though, the median scores fell well within the normal range, and the quartiles were similar. At 24 months corrected age, a decrease of 5 points in cognitive scores was observed. Consequently, cognitive scores were lower than the MDI, but this difference was not significant. The BSID-II MDI includes a considerable number of language items, making a direct comparison with the Bayley-III cognitive score alone less representative of the broader mental development construct assessed by the MDI [3, 17]. Jary et al. demonstrated that the CB-III score correlates more strongly with the MDI than the cognitive score alone [17]. To address this, we also used the CB-III score, which yielded analogous results and provides a more comprehensive measure of cognitive development.

The rates of abnormal cognitive outcomes were similar or even higher in the cohort tested with the Bayley-III German norms compared to the cohort assessed with the BSID-II. It is therefore unlikely that children with cognitive or language developmental delay will be missed by the Bayley-III German norms.

Referring to the motor outcome, we observed higher scores with the Bayley-III German norms until 24 months corrected age. At 12 months, there was a 14-point difference between infants tested with the BSID-II and those assessed with the Bayley-III German norms, which is a larger gap than described in previous studies [6, 18]. Nonetheless, this difference in scores was reduced to 6 points at the corrected age of 24 months, primarily due to an improvement of BSID-II scores from 12 to 24 months in our cohort. This advance of the BSID-II PDI with increasing age is also reflected in the rates of abnormal motor development. While at 12 months corrected age, 35% of the BSID-II cohort had a motor delay and 11% had an impairment, at 24 months corrected age these rates were 15% and 6%, respectively. 63% of the BSID-II cohort who appeared at both time-points and had a motor delay at the first assessment achieved normal scores at 24 months corrected age (result not shown). However, in children tested with the Bayley-III German norms, rates were relatively constant from 12 to 24 months corrected age, and at the latter time-point comparable to the rates of the BSID-II cohort. This leads to the assumption that there are children who have slight abnormalities in the BSID-II at the age of 12 months, which may be missed by the Bayley-III German norms, but who go on to develop well in toddler age. Nonetheless, motor delay is more prevalent in former very preterm infants at preschool age, and it is therefore important to monitor their motor development long-term [19, 20].

Even though revising quantitative neurodevelopmental assessments is necessary due to the Flynn effect, it poses problems for research. Comparing studies that use different versions of one test or planning longitudinal studies that include children tested beyond the introduction of a new version is precarious. However, our study shows that in German-speaking countries, it is scientifically justifiable to include children who were tested with the BSID-II and Bayley-III German norms. In this case, we recommend using the CB-III score to account for the language items used for the MDI.

Yet, with the introduction of the fourth edition of the Bayley Scales the difficulty in presenting the outcome of large cohorts in longitudinal studies will increase. It will be necessary to (1) monitor any unexpected results; (2) wait for population-specific norms; and (3) consider the selection of the standardisation population before routine clinical use and equating results with the BSID-II or Bayley-III in outcome studies. By continuing to use the Bayley-III German norms until the availability and validation of German norms for the Bayley-IV, clinicians can ensure they are utilizing a robust, well-understood assessment tool. Even though statistically significant differences in scores were observed between the BSID-II and the Bayley-III cohorts, developmental delays do not appear to be underrepresented. Thus, despite the observed differences, the Bayley-III German norms seem to offer a reliable means of assessing neurodevelopmental outcomes, with minimal risk of overlooking developmental delays.

In this study, we included a large number of very preterm infants with assessment of neurodevelopmental outcome at 12 and/or 24 months corrected age. Infants were treated and assessed at the only Neonatal Intensive Care Unit in the geographical region of Tyrol, which has a large catchment area, minimising selection bias.

A limitation of our study is, that children were not concurrently tested with both the BSID-II and the Bayley-III. A direct comparison of the BSID-II and Bayley-III German norms within the same patient cohort was not feasible due to practical constraints. Indeed, children tested with the Bayley-III did exhibit a better short-term outcome (Table 1), potentially reflecting differences in medical practices over time. Specifically, Apgar scores at 5 minutes were higher in the Bayley-III cohort. Additionally, the need for catecholamines was reduced, but the Bayley-III cohort experienced longer durations of CPAP therapy, likely reflecting the use of less invasive surfactant administration techniques, which reduced the need for invasive mechanical ventilation. Notably, the incidence of a persistent ductus arteriosus was lower in the Bayley-III cohort, potentially due to the introduction of prophylactic paracetamol treatment during the early neonatal period. Furthermore, there was a significant reduction in the prevalence of retinopathy of prematurity, underscoring improvements in neonatal care. By adjusting for these variables, we aimed to isolate the effects of the developmental assessment tools and minimize biases from evolving clinical practices.

Further, assessments were conducted by three trained psychologists, one of whom participated at both time points. To ensure consistency, testing procedures were standardized through mutual training. Studies have demonstrated high inter-rater reliability for both the BSID-II and Bayley-III, with ICCs ranging from 0.76 to 1.0, suggesting minimal influence of tester variability on the results [21–23].

Moreover, the relatively high Bayley scores within the normal range observed in our cohort of very preterm infants raise questions about the factors contributing to these outcomes. Possible explanations may include early interventions, such as physical therapy, provided to infants identified with developmental concerns at an earlier age. Nonetheless, without a control group for comparison, it is challenging to attribute these outcomes solely to interventions or other factors.

With the upcoming availability of Bayley-IV German norms, future research should compare the Bayley-III and Bayley-IV within the same cohort to assess equivalence and potential differences in outcomes. This will ensure the transition between versions does not obscure key developmental information. Additionally, validating these findings with longitudinal data across diverse German-speaking cohorts will confirm the Bayley-IV’s applicability and reliability in different clinical settings. Future studies should also focus on evaluating the predictive value of the Bayley-III German norms for long-term neurodevelopmental outcomes, such as school performance and cognitive abilities, to better understand its utility as a screening and prognostic tool. Evaluating the predictive validity of the Bayley-III German norms is essential to establish its utility as a prognostic tool. If equivalence between the Bayley-III and Bayley-IV is demonstrated, this would support the assumption that the Bayley-IV retains similar predictive capabilities, facilitating its early adoption in clinical practice with confidence.

## Conclusion

To conclude, higher scores are achieved with the Bayley-III German norms compared to the BSID-II at 12 months corrected age. However, these differences in the median scores do not appear to be clinically relevant and diminish by 24 months corrected age. Importantly, the comparable rates of abnormal neurodevelopmental outcomes between the two scales, particularly at 24 months, suggest that the Bayley-III German norms are effective in identifying very preterm infants in need of intervention. These findings support the continued use of the Bayley-III German norms in clinical practice as a reliable alternative to the BSID-II. However, with the upcoming introduction of the Bayley-IV, the difficulty of presenting outcomes in large longitudinal studies will increase. Clinicians should continue using the Bayley-III German norms until the availability and validation of the Bayley-IV German norms, ensuring consistency in developmental follow-up and minimizing the risk of overlooking developmental delays.

## Supporting information

S1 File (DOCX)

## Abbreviations

Bayley-III: Bayley Scales of Infant and Toddler Development, third edition
BSID: Bayley Scales of Infant Development
BSID-II: Bayley Scales of Infant Development, second edition
CB-III: combined Bayley-Score
MDI: mental developmental index
PDI: psychomotor developmental index
SD: standard deviation
US: United States
