# Supplementary material for: Comparative analysis of developmental outcomes in very preterm infants: BSID-II versus Bayley-III German norms
Source: PLoS One. 2025 Jan 27;20(1):e0318263. doi: 10.1371/journal.pone.0318263 (PMC11771884; doi:10.1371/journal.pone.0318263)
Supplement: S1 File — (DOCX) [file pone.0318263.s001.docx]

# **Supplementary material**

Maternal data included maternal age, achievement of university entrance qualification, antenatal steroid use for fetal lung maturation, prolonged prelabour rupture of membranes, smoking during pregnancy, and mode of delivery. Neonatal data included gestational age in weeks, birth weight in g, sex, Apgar scores, use of surfactant, need for catecholamines, diagnosis of early and late onset sepsis, bronchopulmonary dysplasia, postnatal steroid use for bronchopulmonary dysplasia (hydrocortisone or dexamethasone), patent ductus arteriosus, necrotizing enterocolitis, retinopathy of prematurity, diagnosis of intraventricular haemorrhage or periventricular leukomalacia, duration of ventilation in hours and duration of continuous positive airway pressure in days.

Gestational age was assessed by obstetrical diagnostic estimation and postnatal clinical examination using the modified Ballard score (1). To classify infants as small for gestational age, growth charts developed by Fenton et al. were used (birth weight < 10^th^ centile for sex and gestational age) (2). Bronchopulmonary dysplasia was defined as need for supplemental oxygen for at least 28 days (3). All infants received echocardiography after at least 72 h of life to identify a patent ductus arteriosus. Diagnosis of patent ductus arteriosus was given if echocardiographic criteria were fulfilled (4). Necrotizing enterocolitis was defined according to Bell’s criteria (5). Retinopathy of prematurity was classified according to the International Classification of Retinopathy of Prematurity (6). Head ultrasound was routinely performed in all infants. Intraventricular haemorrhage and periventricular leukomalacia were defined using the method of Papile et al. and de Vries et al. (7, 8).

1. Ballard JL, Novak KK, Driver M. A simplified score for assessment of fetal maturation of newly born infants. J Pediatr. 1979;95(5 Pt 1):769-74.

2. Fenton TR, Kim JH. A systematic review and meta-analysis to revise the Fenton growth chart for preterm infants. BMC Pediatr. 2013;13:59. doi: 10.1186/1471-2431-13-59.

3. Jobe AH, Bancalari E. Bronchopulmonary dysplasia. Am J Respir Crit Care Med. 2001;163(7):1723-9.

4. Alagarsamy S, Chhabra M, Gudavalli M, Nadroo AM, Sutija VG, Yugrakh D. Comparison of clinical criteria with echocardiographic findings in diagnosing PDA in preterm infants. J Perinat Med. 2005;33(2):161-4.

5. Bell MJ. Neonatal necrotizing enterocolitis. N Engl J Med. 1978;298(5):281-2.

6. The International Classification of Retinopathy of Prematurity revisited. Arch Ophthalmol. 2005;123(7):991-9.

7. Papile LA, Burstein J, Burstein R, Koffler H. Incidence and evolution of subependymal and intraventricular hemorrhage: a study of infants with birth weights less than 1,500 gm. J Pediatr. 1978;92(4):529-34.

8. de Vries LS, Eken P, Dubowitz LM. The spectrum of leukomalacia using cranial ultrasound. Behav Brain Res. 1992;49(1):1-6.
